# Looking back to look forward: a review of human resources for health governance in South Africa from 1994 to 2018

**DOI:** 10.1186/s12960-020-00536-1

**Published:** 2020-11-26

**Authors:** Manya Van Ryneveld, Helen Schneider, Uta Lehmann

**Affiliations:** 1 School of Public Health, University of the Western Cape, Robert Sobukwe Road, Bellville Cape Town, 7535 South Africa; 2 Health Services To Systems Research Unit, South African Medical Research Council, Cape Town, South Africa

**Keywords:** Health human resources, Governance, Stewardship

## Abstract

While South Africa has had a fairly consistent record of producing national-level strategic plans for human resources for health in the past 25 years, the country continues to face major problems of affordability, availability, distribution and management of its health workforce. There are several factors contributing to the state of health human resources in the country, but problems with governance stand out as one area requiring further research, analysis and critique. This paper presents a retrospective analysis of the historical patterns in national health human resources governance in South Africa, based on a desktop policy review spanning 25 years after democracy. The authors took a multi-pronged, iterative approach, reviewing policy documents alongside grey and published literature. This led to a timeline showing key legislation, relevant health system and human resource policies, interventions, reviews and evaluations from 1994 to 2018. The review identified three distinct periods that help to characterise the terrain of human resources for health governance over the concerned 25 years. Firstly, a foundational period, in which much of the constitutional and legislative groundwork was laid. Secondly, the HIV epidemic period, which presented a major disruption to the development of system wide governance interventions and improvements. Thirdly, the launch of National Health Insurance discussions and policy processes, which signalled a gradual return to a comprehensive systems focus in line with the founding principles of the first period. Using this periodisation, as well as a conceptual framework of health human resources governance functions based on international literature, the authors argue that South Africa has experienced both progress and challenges in human resources for health governance. This has affected the successful implementation of its policy and strategic planning over the past 25 years. Good governance for human resources for health requires capable stewardship, underpinned by an appropriate mix of technical and administrative skills and explicit political support. The review findings suggest that strengthening human resources for health governance roles, including fostering purposeful stewardship by the National Department of Health, may be key to shifting the terrain in the availability and performance of South Africa’s health workforce going forwards.

## Background

A global shortage of 15 million health workers is predicted by 2030, with a major concentration of extreme shortages expected in the African region [1]. With renewed commitments towards universal health coverage (UHC) being made globally, addressing the state of human resources for health (HRH) will be fundamental for any government hoping to honour their commitments. While the importance of HRH development in health systems research is recognised, the practice of governance in relation to the field of HRH policy and planning is poorly established in many settings [2]. Structures and capabilities for HRH governance are often under-developed and lacking in political support [3].

It is widely acknowledged that HRH development requires coordination of multiple stakeholders and levels of government [3–5]. Developing comprehensive national HRH policies is just one of the steps towards enabling good governance of, and bridging gaps in, HRH. The capacity required to do so lies principally with national ministries of health, who are well-positioned to play important oversight and stewardship roles. However, a review of HRH units in the ministries of health of 26 countries in the World Health Organization’s (WHO) African region reported that most country-level HRH units are “poorly structured, not fit for purpose and lack the ability to influence policy directions” (4, p. 2). Another study showed that, in 2015, only 36% of countries in the African Region had national-level HRH policy [3].

South Africa (SA) is among those countries with national-level HRH strategic plans, however it continues to face profound health workforce challenges, many of which stem from a lack of effective HRH governance and high-level stewardship of the implementation of HRH plans. SA spends 8% of its GDP on health and 63.4% of health expenditure in the public sector goes towards health personnel [6]. Despite numerous HRH-related interventions over the last 25 years, the country continues to face major problems of affordability, availability, distribution and management of its health workforce [6]. Health workers are poorly distributed throughout the country, with stark contrasts along rural and urban, and public and private sector lines. Overburdened and poor working conditions, fractious labour relations and punitive organisational cultures exacerbate the problem and are often seen as the frontline push factors that contribute to maldistribution and brain drain.

This paper presents a desktop review and historical analysis of national-level HRH governance and stewardship in SA since the advent of democracy in 1994, through the lens of four key strategic HRH functions and the national-level stewardship capacity required to fulfil these functions. The analysis covers three distinct periods that characterise the historical terrain of the broader health system in South Africa and its impact on HRH governance—the foundational period directly after 1994, a period dominated by the HIV/AIDS epidemic and the more recent shift in focus towards National Health Insurance. A retrospective look at trends and tendencies in HRH governance is an important exercise in consolidating the lessons that can be learnt from the past for policies and plans going forward. This is especially relevant in South Africa, as significant reforms towards National Health Insurance (NHI) and Universal Health Coverage (UHC) are being advanced, a new 2030 national HRH strategy and 5-year plan is launched, and the impacts of the Covid-19 pandemic are addressed.

### Conceptual framework

**Fig. 1:**
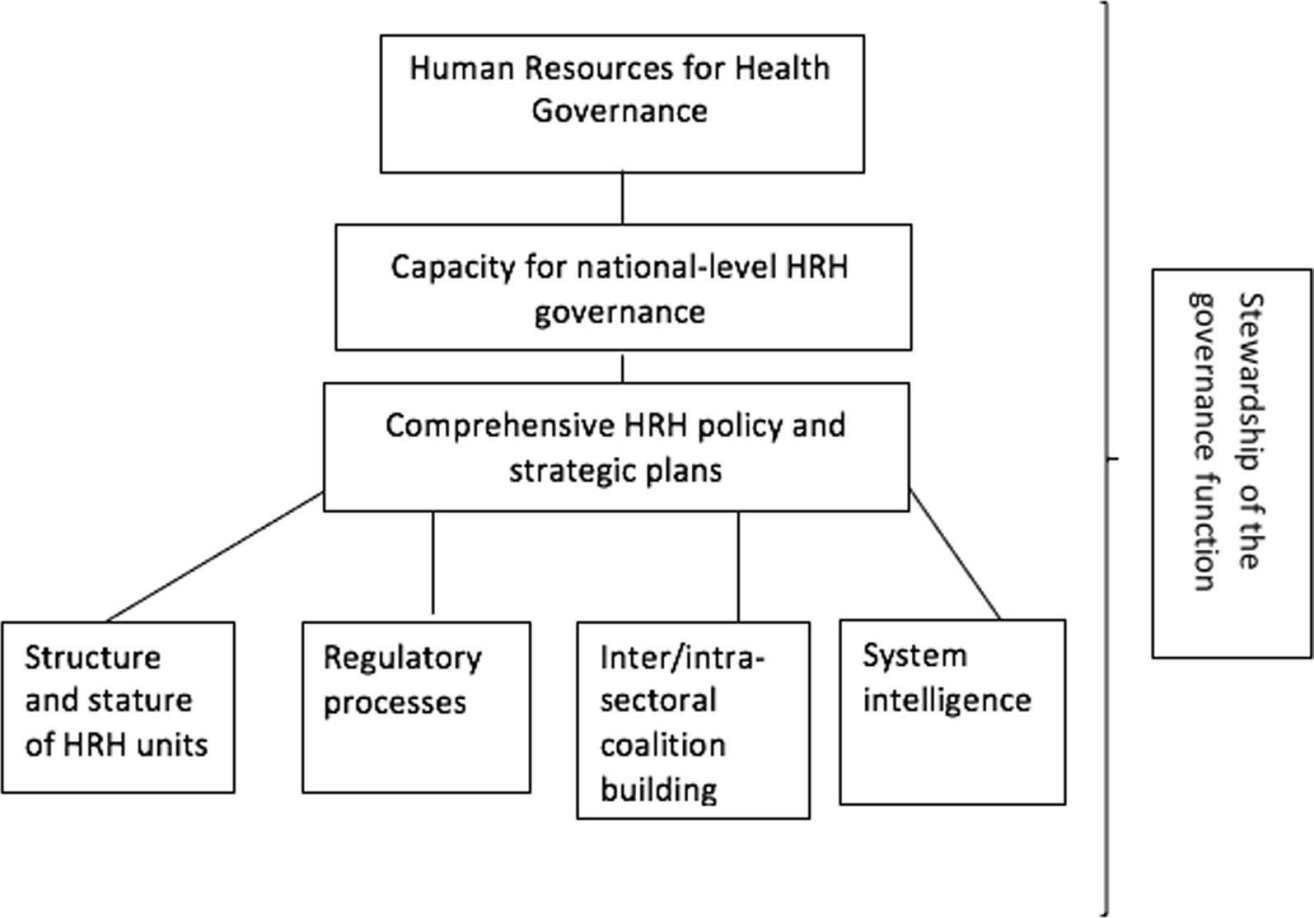
Conceptual framework of HRH governance, adapted from Nyoni and Gedik [4] and Afriye et al. [3]

To support deeper analysis of the data, a conceptual framework to describe the interaction between the concept of governance and the stewardship function within governance was developed (Fig. 1), based on work done for the WHO Africa Region by Nyoni and Gedik [4] and Afriye et al. [3]. This framework takes the HRH governance function as the overarching concept, understanding it as being steered, in the first instance, through national-level HRH processes and structures. The HRH function at national level includes not only well formulated, implementable national-level HRH policy, it should also be enabled to perform its governance function through (i) an appropriate structure and stature of the HRH unit and its programmes within the ministry of health; (ii) strong coordination with regulatory structures and professional councils, (iii) inter- and intra-sectoral coalition building across the levels of government and with relevant departments such as Higher Education and Finance; and (iv) access to and coordination of good-quality health workforce intelligence for decision-making—through routine data collection as well as monitoring and evaluation systems. This requires a strong focus on stakeholder engagement and management across levels and sectors of government, regulatory structures and the professions. In addition, it should be recognised that these components all require political support and leadership and involve a large degree of relational and actor management. Together, these elements make up the stewardship role, which is the (sometimes intangible) cross-cutting component of governance that acts as the “glue” that holds the other planning and implementation functions together.

## Methodology

### Gathering and consolidating resources

**Fig. 2:**
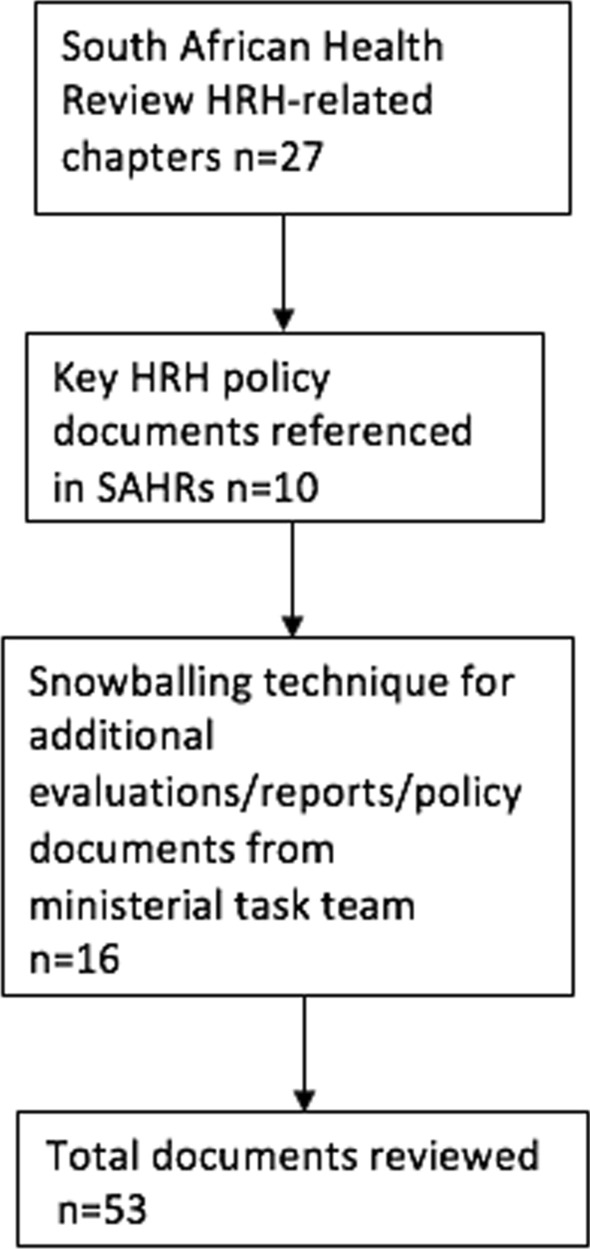
Flowchart of literature and policy documents reviewed

For this review, the authors employed a multi-pronged, iterative methodology, reviewing both literature and a selection of relevant policy documents (see Fig. 2). The first step was to generate a historical overview of HRH policy in SA using the South African Health Review (SAHR)—an annual publication of the Health Systems Trust [7], which documents and comments on the state of SA’s health system and has been published annually since 1995. Twenty-seven HRH-related chapters of the SAHR were included. Where the chapters referenced national-level HRH policy documents, these were sourced, read and summarised. Scanning of references also led to identification of other relevant literature, supplementary HRH policy documents, the National Department of Health (NDoH) annual reports, and a number of HRH-related evaluations conducted at the national level. Relevant historical information was captured on a timeline (see Additional file 1) and an annotated bibliography of national-level HRH interventions and policies was developed. Finally, a version of the review was submitted as an input to a sub-group on Governance and Leadership of the Ministerial Task Team formulating the latest HRH strategy, which identified additional key events for the timeline as well as additional documentary sources to review.

The review findings are structured into six sections. The first two lay out a contextual overview of HRH in South Africa and the historical periodisation of HRH governance and planning. Then, findings regarding each of the four elements underpinning HRH governance, taken from the conceptual framework, are discussed in the remaining sections.

## Unpacking 25 years of HRH governance in South Africa

### Contextual overview

While the number of health professionals in SA is higher than most sub-Saharan African countries, SA still faces a crisis of health worker shortages, particularly in the public sector and in rural areas [8, 9]. This is exacerbated by poor distribution and retention due to undesirable work environments, increasingly overburdened staff with shrinking staff establishments, moonlighting and unmanaged remuneration for work done outside of the public sector, an inappropriate skills mix, and the lack of a nationally integrated information system to accurately calculate HRH supply needs, especially at disaggregated levels [9].

SA’s health workforce has a mix of established professional cadres, with nurses being by far the largest category. Alongside these professionals are large numbers of community health workers providing outreach, and a relatively small number of mid-level cadres. The introduction of mid-level cadres such as the pharmacy assistant and clinical associate began in the early 2000s, but has faced challenges regarding funding, effective deployment, integration with other health professionals and recognition of their scope of practice by the relevant regulatory bodies [10].

As legislated in the National Health Act of 2003 and the Labour Relations Act of 1995, the various health professions are governed by their respective statutory bodies, who play an important role in regulating the training, registration, community service, continuing professional development and dispensing practices of all health professionals. The function of health professions’ education and training is shared across a number of players including university faculties of Health Sciences, Academic Health Complexes, nurse training institutions and the Bi-Ministerial Join Health Sciences Education Committee (JHSEC).

At the national level, reporting to the Minister and the Director-General of Health, there is a Deputy Director-General who oversees the budget programme for hospitals, tertiary health services and workforce development. HRH and responsibilities regarding medium to long-term HR planning, development and management, as well as the office of nursing, belong to this programme. Provincial departments of health are responsible for implementing and funding the national HRH strategy at the provincial level, through advertising and filling vacancies using their provincial HR plans.

### Historical overview

Broadly speaking, HRH governance from 1994 to 2018 can be divided and characterised into three key periods. The first period was a foundational phase immediately post-1994, which saw a major overhaul of policy and legislation and established the frameworks for broader health system transformation, including HRH, in a democratic SA. The focus of this period was one of tackling the racialisation and fragmentation of health care facilities and services, and a shift towards the primary health care (PHC) approach [11]. In 2001 the Department of Health released an unofficial, but wide-ranging and substantially detailed report from the first Ministerial Task Team on HRH—the “Pick Report”. Although technically an unofficial document, it laid important groundwork for HRH thinking in the context of PHC, addressing issues of low production, maldistribution and under-supply of suitable health professionals [12]. The HRH implications of a newly formulated PHC package were outlined in terms of the “staffing gap” and the need for revised scopes of practice and the introduction of mid-level workers. An emphasis was placed on the need for skill audits and surveys as a fundamental component of HRH planning, monitoring and evaluation of progress.

The second period spans the decade between 1999 and 2009, during which SA’s health system faced a rapidly unfolding and catastrophic HIV epidemic associated with a massively contested HIV policy terrain. This had a major and multi-faceted impact on the health workforce, firstly, in the rapid increases in workloads, despair and demotivation in the face of death and dying, secondly, in the morbidity and mortality from HIV within the health workforce itself and thirdly, in the under-prioritisation of systems issues (including HRH policy and key health legislation) in lieu of a programmatic focus on HIV [13].

In 2004, the long-awaited National Health Act was finally signed into law, giving the Minister of Health power to create regulations, institutional capacity and governance structures to ensure a well-trained, equitably distributed and retained health workforce for SA. The National Health Act was thus an important legislative step, placing stewardship for HRH within the remit of the minister and national government. In 2006 the first national HRH strategic plan was released [14]. A national Nursing Strategy was also released in 2008 [15]. The 2006 HRH strategy was presented as a comprehensive national human resource plan in the context of the recently passed National Health Act, guiding the development of provincial HR plans and serving as a reference point for the private sector and for education and training institutions.

However, the impact of the Act and the strategic plan was limited by a number of factors, including the continued programmatic focus on anti-retroviral (ARV) rollout, various crises in nursing education and training, health worker strikes over the implementation of Occupation Specific Dispensations, and broader austerity measures brought about by the 2008 financial crisis. HRH policy and planning floundered in a health system shaped by vertical, disease-specific programmes and a legislative and policy vacuum regarding the delegation of responsibility for PHC between spheres of government prior to the finalisation of the National Health Act [13]. Many of the important systems-oriented aspirations that were laid out in the policy documents of the early democratic years took a backseat in the face of this.

The third phase, from 2009/2010 signalled the start of a gradual return to a comprehensive systems focus in line with the founding principles of the first period, with the release of the Green Paper for National Health Insurance (NHI) in 2009 and the launch of a PHC Re-engineering Strategy in 2011. This coincided with the release of the second national-level HRH strategic plan—the HRH Strategy for the Health Sector 2012/2013–2016/2017 [16] and a Nursing Education and Training strategy [17]. However, the legacy of the previous decade’s vertical, disease-oriented response remained embedded in the budget programme structures at national level and the extent of political support for HRH prioritisation was not explicit, despite the renewed systems focus [18]. With little attention paid to continuity of ideas or pause for evaluation and assessment of what was already in place, HRH reforms implemented in this period were piecemeal and specific [16, 19].

This periodisation exposes a distinct lack of continuity between approaches to HRH planning exercises over the past 25 years. The plans themselves respond predominantly to the varying political and legislative environments of their time (respectively—post-Apartheid transformation, the passing of the National Health Act, and the introduction of NHI and PHC re-engineering). Secondly, despite plainly acknowledging many human resource failings, such as poor distribution, brain drain, overburdening and poor working conditions, and being written with actionable goals in the short, medium and long term, implementation of these plans has been undermined by a lack of capacity and failures in other dimensions of the governance and stewardship function—such as the structure and stature of the national HRH unit, regulatory processes, inter- and intra-sectoral partnerships and coordination, and the access to and use of system intelligence. We examine these dimensions in the remainder of this paper.

### Structure and stature of the national HRH unit

The existence of enabling structures for HRH is central to the overall success of policy implementation. While the framework and structures for HRH governance in SA were established by the National Health Act, performance has been undermined by an under-prioritisation of their functions. The position and stature of the HRH unit within the National Department of Health (NDoH) is a case in point. As the 2003/2004 SAHR pointed out, the Directorate for Human Resource Development has continued to face “high staff turnover rates and a number of vacant posts” undermining the in-house capacity for HRH governance (20, p. 300).

**Fig. 3:**
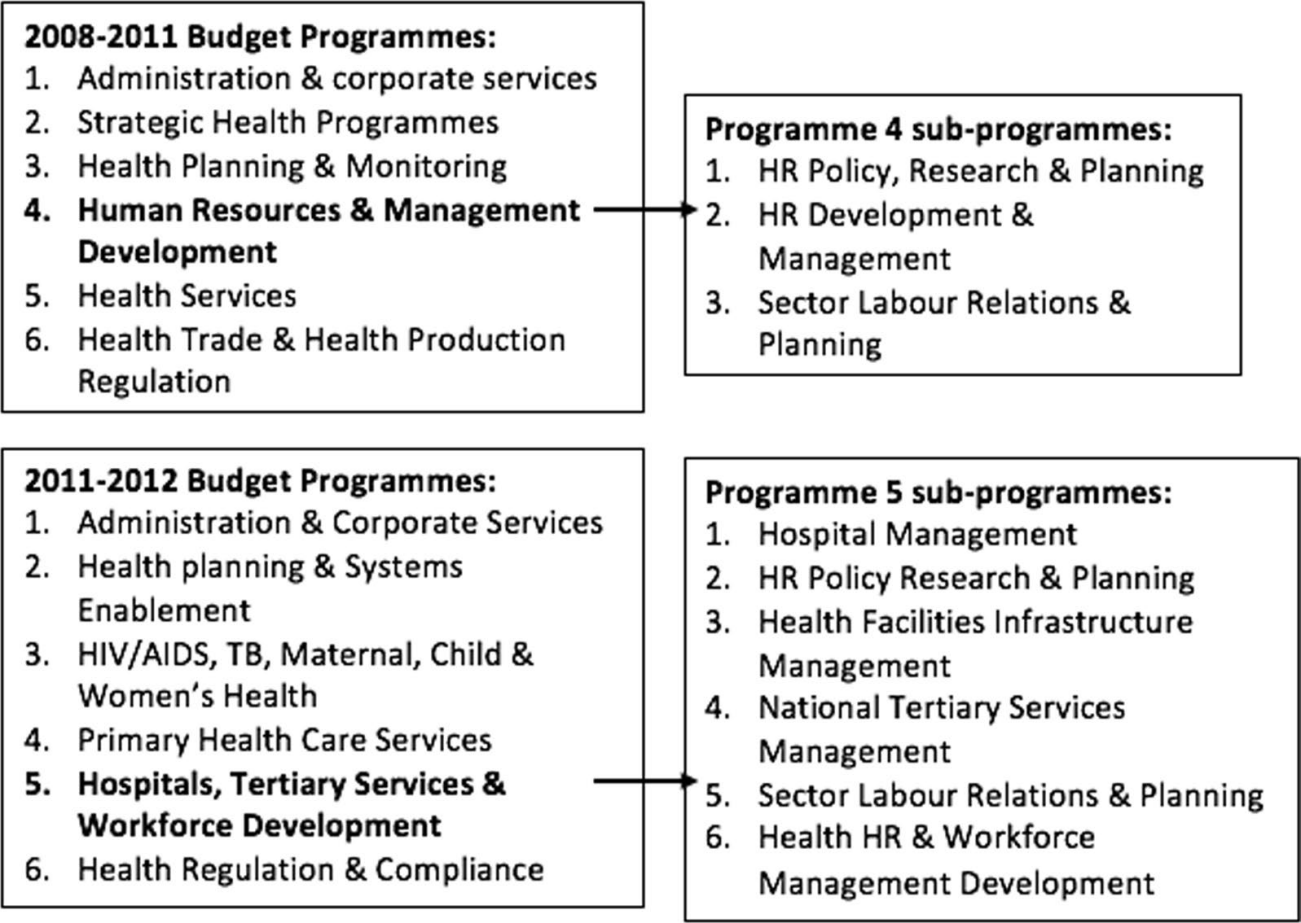
Changes to budget programme structure for HRH at the National Department of Health

Information on the composition of the national HRH unit was obtained from the NDoH’s annual reports from 2008 onwards, when new organisational structure and budget programmes were introduced (Fig. 3). Between 2008 and 2011, the HRH unit had its own budget programme, with three sub-programmes, entirely dedicated to HR management and development. However, in the 2011/2012 financial year, the department underwent restructuring to improve alignment with national health priorities and manage new nationally controlled conditional grants [18]. HRH no longer had its own budget programme, but instead became a sub-programme of a programme that also included hospital management, health facilities infrastructure and national tertiary services. In 2012/2013, after calls for a Chief Nursing Officer to be established in the department, the office of Nursing Services was introduced and included in this budget programme.

As of the 2017/2018 financial year, human resource development and nursing services remain part of this larger programme, along with hospital and tertiary health services, trauma, violence, emergency and forensic pathology medical services, and health facilities infrastructure planning. This suggests a continued dilution of the HRH unit in favour of programmatic approaches and a budget structure designed on the basis of disease-specific conditional grants. Without an in-house HRH unit at national level that functions transversally, is well resourced and politically well-regarded, the capacity to implement even the best strategic plans is undermined.

### Regulatory processes

A key function of HRH governance is ensuring appropriate regulation of the health workforce. In this regard, SA has relatively well-developed processes, involving a number of health professionals boards representing nurses, medical and dental professionals, pharmacists, rehabilitative professionals, traditional health practitioners and allied health workers. The Health Professions Council of South Africa (HPSCA), the South African Pharmacy Council (SAPC) and the South African Nursing Council (SANC) are the three bodies representing the majority of health professionals in both the public and private sector (Table 1). Community health workers do not as yet have a regulatory board or professional council.

**Table 1:** Statutory bodies regulating health professions in SA

Health profession	Statutory body
Medical doctors and dentists	Medical and Dental Board of the Health Professions Council of South Africa (HPCSA)
Professional nurses, enrolled nurses and nursing assistants	South African Nursing Council (SANC)
Pharmacists and pharmacy support personnel	South African Pharmacy Council (SAPC)
Rehabilitation professionals (OTs, physiotherapists, speech therapists)	Professional Boards of the HPCSA
Environmental health practitioners	Professional Board of the HPCSA
Traditional healers	Interim Traditional Health Practitioners Council
Community health workers	None

These bodies, many established in the colonial and apartheid eras of a rule bound, hierarchical and militaristic culture in health care [21], are responsible for setting the education and training standards that govern the professions, and regulating their registration, professional conduct, continuing professional development, community service, internship and dispensing practices. This is done in accordance with health-related legislation such as the National Health Act (2003), the Health Professions Act (1974), and the Nursing Act (2005), and wider legislation such as the Labour Relations Act (1995) and the Skills Development Act (1998).

Despite this relatively strong framework, poor governance within and across the various regulatory bodies, as well as weak coordination with the HRH unit at NDoH, have resulted in serious regulatory bottlenecks. This has led to a cumbersome, reactive and often punitive regulatory environment, described as impeding health professionals’ ability to sit exams, register or practise effectively and impacting on the absorption of much needed foreign health professionals, new cadres or community service professionals and interns into the system [22].

Furthermore, regulatory processes have not aligned well with national HR processes and policies. For example, the introduction of mid-level Clinical Associates (CAs) in 2011 has been undermined by HPCSA rules that disallow them from prescribing basic medication, even though this was envisioned as being part of their scope of practice and they have been trained to do so [10]. As Doherty et al. have pointed out, health professionals who feel threatened by the introduction of CAs can use regulatory issues as a way of “undermining their work or curtailing their usefulness” (10, p. 835), signalling regulatory capture by the professions.

The alignment between regulatory bodies and national policy, requires strong governance capacity in both NDoH and the professional boards to ensure that any contestation over new policy can be dealt with timeously and effectively. However, the two major professional bodies, the HPCSA and the SANC have themselves experienced significant governance weaknesses over the past 25 years [23]. In 2015 the HPCSA came under investigation for administrative irregularities, mismanagement and poor governance [22]. Similarly, the 2012/2013 Nursing Education and Training Strategy highlighted “concerns about the SANC’s governance, notably its sub-optimal leadership and stewardship which has impacted on its professional governance role” (17, p. 24).

### Inter/intra-sectoral coalition building

HRH governance involves working with multiple stakeholders, and requires good coordination, seamless policy dialogue, a shared vision and underlying political will [4]. The 2004 Joint Learning Initiative on Health Human Resources emphasised the need for both strong collaborative approaches and political traction, bringing together stakeholders from the health sector, government and beyond [24]. The responsibility for building and coordinating these relationships lies at the heart of the HRH stewardship function at national level, addressing a complex array of interfaces: between national and sub- national spheres of government; between health and other government departments such as Higher Education, Finance, Public Service and Administration, and Labour; and between government and professional regulatory and higher educational institutions. A lack of strong relationships and channels of bi-directional communication between these different stakeholders has major implications for policy implementation [25].

Health professions’ education (HPE) is perhaps the most affected by fragmented relationships across sectors and the lack of a shared vision. A case in point is the crisis in nursing education, which has suffered from a lack of inter-sectoral coordination between the NDoH, Department of Higher Education and Training (DHET) and the SANC [26]. This led to extensive delays in gazetting government regulations for accrediting a new nursing curriculum and the training institutions that will teach it, threatening to curtail the already low production of nurses for the country [27]. Finding a resolution to these reforms over the years has proven difficult, with research citing weaknesses in the leadership and governance capacity of the main nursing institutions to implement appropriate policy [28].

Without strong coordination between the professional councils, NDoH, DHET and Higher Education Institutions (medical schools, nursing colleges, etc.), responding to changing HRH needs through, for example, developing new curricula, introducing new mid-level cadres or adjusting student intake to encourage rural retention, is difficult. The 2018 Academy of Science of South Africa (ASSAf) Consensus Study reviewed the state of HPE and put forward a number of concrete recommendations for its transformation in light of both NHI and UHC, as well as the stark reality of health professional shortages [29]. Recommendations include revisiting student selection, training and support to improve practice in rural and underserved areas and restructuring curricula for more inter-professional education and collaborative practice, to name a few.

The ASSAf recommendations were underpinned by a call for multi-stakeholder and multi-sectoral approaches, and strong and appropriate governance structures, all of which must be guided by a spirit of collaborative action. To date, a key mechanism for tackling issues of inter-sectoral collaboration in HPE has been the Bi-Ministerial Joint Health Sciences Education Committee (JHSEC). Established in 2014, it involves both the DHET and the NDoH, with National Treasury as a “participating member”, and was set up to “co-ordinate and align strategy, policy and financing in health sciences education” [29]. However, as the ASSAf report points out, the JHSEC has not functioned optimally, while many crises in health professions education remain unresolved. This demonstrates that the mere existence of a collaborative committee is not sufficient to solve complex issues involving inter-sectoral action. A key recommendation in the ASSAf report was the strengthening of JHSEC as a governance structure and “building a joint vision amongst its members” (29, p. 199).

### System intelligence

With respect to HRH, we define system intelligence as the information that contributes to effective HRH planning—in other words, routine data collection and monitoring and evaluation—as well as the intelligent deployment and usage of this information to improve policy, planning and implementation. As stressed by all of the HRH strategies to date, the HRH information system is not entirely suitable for evidence-based planning and management of HRH. Information comes from disparate sources such as PERSAL—the system used to manage personnel and salaries across the public sector in SA. This database is used as a proxy information system for the public sector health workforce. Given that PERSAL is primarily a pay-roll system designed to manage finances and salaries, it is not well-suited to HRH planning and is often poorly updated [30]. Other databases for health workers in the private sector, such as the registries of the professional councils, are also unreliable, often containing health professionals who are no longer working or have left the country [31].

Other avenues for generating information for HRH planning remain unreliable; the lack of good-quality information is an issue that plagues multiple facets of SA’s health system [23]. Evaluations of policy implementation should play an important role in generating valuable HRH planning information. This is particularly so when HRH is in crisis and the purpose of strategic planning is to address chronic underlying difficulties such as poor distribution and low production [32]. In SA there have been no formal evaluations of the implementation of any of the country’s official HRH plans to date. While national and provincial governments have processes for internal progress and performance auditing, as well as evaluation systems set up by the Department of Monitoring and Evaluation, external evaluation to generate feedback on the implementation of the plans themselves has not been done. In general, provincial-level HRH plans and evaluation mechanisms are not uniformly understood as sources of system intelligence or workforce planning exercises, but merely as auditing requirements [16]. Where evaluations of HRH-related policy have been undertaken, they have largely been in the context of major health systems crises such as the Integrated Support Teams reports (which documented the crisis of provincial health departments’ budgetary shortfalls as a result of failings in both political and administrative leadership), or in response to specific crises such as the maladministration of the HPCSA. Using evaluations only to respond to crises, while important for accountability, undermines their usefulness in generating systematic and continuous feedback information that can contribute towards further improvement of policy and improved performance, management and distribution of the health workforce.

There is also a lack of comprehensive evaluation of the various HRH-related interventions and initiatives. While some, such as community service have been evaluated by independent researchers [33, 34], several major initiatives have never been comprehensively evaluated. A case in point is the Occupation Specific Dispensation, which was a costly intervention aimed at financially incentivising health professionals to remain in the public sector. It was delivered as an unfunded mandate to the provinces, with very little guidance or vision for implementation [16]. The result was a huge financial drain on the provinces, exacerbation of already fractious labour relations and major public sector strikes.

## Recommendations

Based on the key elements of HRH governance outlined in the conceptual framework, it is clear that there are profound weaknesses in (i) the structure and stature of the HRH unit at NDoH; (ii) alignment of the regulatory bodies with national policy; (iii) the relationships and coalitions between the diverse stakeholders and (iv) the availability of the right kind of information for planning and decision-making. We suggest that strong HRH governance, as well as explicit stewardship of that function, is a key attribute in tackling this and, in the case of SA, requires renewal and clarification of the HRH governance priorities at national level.

Firstly, the HRH unit at NDoH needs to be capacitated to perform its governance function. While good HRH strategic plans are an important part of this function, they should aim to support the development of further HRH planning at the lower levels of government, rather than lock sub-national departments into fulfilling plans they cannot realistically fulfil. This requires that strong information and monitoring and evaluation systems are in place to facilitate decision-making closer to the frontline. Secondly, there is a need to understand what else can improve the organisational capacity for HRH governance at national level, in particular what Aragón calls the “intangible software”—the values, norms, informal rules and relationships—that are fundamental to effective actor management and establishing a shared vision [35]. This review clearly demonstrates that good governance requires an appropriate mix of skills and explicit political support—it is not purely a technical and administrative function. Thus, it is imperative that all the relevant actors and skillsets are given appropriate legitimacy and authority in the decision-making arena. Finally, HRH governance is an area in dire need of more research and more attention. This requires addressing the “tendency to conflate ‘management’ with ‘governance’ which are, in fact, very different terms, albeit closely related” (2, p. 2). While HRH management has been fairly comprehensively addressed in the literature, HRH governance remains an elusive knowledge gap. This desktop review has scratched the surface of how poor engagement with national-level governance roles has impacted negatively on HRH policy implementation and more evidence and analysis is needed.

SA has made some progress in addressing its HRH challenges and has been able to produce fairly consistent national-level strategic plans that offer some form of strategic vision. Nevertheless, the landscape of HRH policy and planning is also marked by systemic breakdowns, crisis management, and plugging holes, all of which requires a concerted effort to reverse. Much of this is symptomatic of a lack of continuity between HRH strategic plans, a lack of capacity for stewardship of HRH policy at national level, and major issues with inter-sectoral and multi-stakeholder collaboration. The findings of this review and analysis using the conceptual framework demonstrate that without strong political support for, and stewardship of, the HRH governance function at the national level, the effectiveness of HRH policy and planning will continue to be undermined. Addressing this will be fundamental to bridging SA’s policy–implementation gap, especially with regards to the proposed NHI and UHC policy reforms.

## Supplementary information

**Additional file 1:** Human Resources for Health Timeline. A timeline showing legislative and policy developments in Human Resources for Health in South Africa between 1994 and 2018.

## Data Availability

Not applicable.

## Abbreviations

ASSAf: Academy of Science South Africa
ANC: African National Congress
ARV: Anti-retroviral
CA: Clinical Associates
DHET: Department of Higher Education and Training
DPSA: Department of Public Service and Administration
HPSCA: Health Professions Council of South Africa
HPE: Health Professions’ Education
HRH: Human resources for health
JHSEC: Joint Health Sciences Education Committee
NDoH: National Department of Health
NHI: National Health Insurance
PHC: Primary health care
SA: South Africa
SAHR: South African Health Review
SANC: South African Nursing Council
SAPC: South African Pharmacy Council
UHC: Universal Health Coverage
WHO: World Health Organization
